# Modulation of Dendritic Cell Activation and Subsequent Th1 Cell Polarization by Lidocaine

**DOI:** 10.1371/journal.pone.0139845

**Published:** 2015-10-07

**Authors:** Young-Tae Jeon, Hyeongjin Na, Heeju Ryu, Yeonseok Chung

**Affiliations:** 1 Department of Anesthesiology and Pain Medicine, College of Medicine, Seoul National University, Seoul, Republic of Korea; 2 Laboratory of Immune Regulation, Institute of Pharmaceutical Sciences, College of Pharmacy, Seoul National University, Seoul, Republic of Korea; KAIST, Graduate School of Medical Science & Engineering, REPUBLIC OF KOREA

## Abstract

Dendritic cells play an essential role in bridging innate and adaptive immunity by recognizing cellular stress including pathogen- and damage-associated molecular patterns and by shaping the types of antigen-specific T cell immunity. Although lidocaine is widely used in clinical settings that trigger cellular stress, it remains unclear whether such treatment impacts the activation of innate immune cells and subsequent differentiation of T cells. Here we showed that lidocaine inhibited the production of IL–6, TNFα and IL–12 from dendritic cells in response to toll-like receptor ligands including lipopolysaccharide, poly(I:C) and R837 in a dose-dependent manner. Notably, the differentiation of Th1 cells was significantly suppressed by the addition of lidocaine while the same treatment had little effect on the differentiation of Th17, Th2 and regulatory T cells *in vitro*. Moreover, lidocaine suppressed the ovalbumin-specific Th1 cell responses *in vivo* induced by the adoptive transfer of ovalbumin-pulsed dendritic cells. These results demonstrate that lidocaine inhibits the activation of dendritic cells in response to toll-like receptor signals and subsequently suppresses the differentiation of Th1 cell responses.

## Introduction

Recognition of pathogen-associated molecular patterns (PAMPs) such as toll-like receptor (TLR) ligands as well as damage-associated molecular patterns (DAMPs) such as high mobility group box 1 (HMGB1) by innate immune receptors leads to the activation of macrophages and dendritic cells [1, 2]. Tissue resident macrophages are known to sense these exogenous and endogenous stimuli to produce immune modulatory molecules such as IL–6, TNFα as well as reactive nitrogen species and reactive oxygen species that can mediate tissue inflammation [3, 4]. On the other hand, activation of dendritic cells by PAMPs and DAMPs not only triggers the production of pro- or anti-inflammatory cytokines, but also induces their migration into lymph nodes and subsequent activation of T cells in an antigen-specific manner [5, 6]. Depending on the types of cytokines and costimulatory molecules expressed by dendritic cells, the interacting antigen-specific T cells can acquire diverse effector functions. In case of CD4^+^ T cells, these effector T cells include Th1, Th2, follicular helper T, Th17 and regulatory T cells, all of which that have unique effector functions in adaptive immune arms [7–9]. Hence, modulation of innate immunity in response to PAMPs and DAMPs can determine the type(s) of adaptive immunity as well as that of innate immunity during inflammation.

Anesthetic agents are widely used to reduce pain and psychological stress during a process involving tissue damage including perioperative practice which can trigger the production of DAMPs by damaged cells as well as PAMPs by invading infectious agents [10]. It is well documented that surgical stress modulates the function of innate immune cells. For instance, surgical stress has been shown to mediate endotoxin hypo-responsiveness by increasing the production of IL–10 while decreasing the production of TNFα [11, 12]. In addition, a number of anesthetics exhibit immune modulatory activity, either by directly acting on immune cells or indirectly by affecting hypothalamic-pituitary-adrenal axis in experimental animals as well as in humans [13, 14]. In general, anesthetics are known to exert immune suppressive activities in innate immune cells. For instance, lidocaine inhibits phagocytic activity, chemotaxis and activation of human neutrophils [15–18]. Similarly, lidocaine suppresses the production of nitric oxide from murine macrophages upon stimulation with lipopolysaccharide (LPS) and IFNγ, possibly through the regulation of voltage-sensitive Na^+^ channel [19, 20]. Furthermore, administration of lidocaine has been shown to inhibit acute lung injury induced by LPS via suppressing the activation of the NF-κB signaling pathway in an animal model of endotoxemia [21]. Similarly, the production of IL–1 and IL–6 as well as the expression of ICAM–1 on activated endothelial cells is down-regulated by lidocaine [22]. These immune suppressive activities of anesthetics can be problematic in patients with tumor or infections since the suppression of immune competent arms would be detrimental in fighting against cancer cells and infectious agents [23].

Lidocaine is the only local anesthetic that is approved for intravenous administration in clinical practice. Lidocaine has an anti-inflammatory property by attenuation of production of pro-inflammatory cytokines which are known to cause inflammatory and neuropathic pain [24]. Systemic administration of lidocaine reduces surgery-induced immune reactions via decreased production of pro- and anti-inflammatory cytokines (IL–6 and IL-1Ra, respectively) during abdominal hysterectomy [25]. Intravenous lidocaine infusion reduces postoperative pain intensity and analgesic requirements in patients undergoing abdominal surgery [26]. Perioperative infusion of lidocaine reduces the incidence of post-mastectomy chronic pain [27]. Of note, lidocaine has been known to induce allergic reactions in humans; while type I or anaphylactic hypersensitivity to lidocaine injection is uncommon [28], allergic contact dermatitis to lidocaine is becoming more prevalent [29]. It seems that the allergic reactions of lidocaine might be associated with a delayed type IV hypersensitivity reaction, which is mediated by antigen-specific T cells. Thus, lidocaine not only affects the production of cytokines from innate immune cells, but also may modulate antigen-specific T cell immunity in humans.

Although the immune-modulatory function of lidocaine has been suggested, it remains poorly understood if lidocaine regulates the activation of dendritic cells and differentiation of antigen-specific helper T cells. In the present study, we aimed to examine the role of lidocaine on the activation of dendritic cells in response to diverse toll-like receptors, and to examine the role of lidocaine on the differentiation of helper T cells mediated by dendritic cells. Our data revealed that lidocaine globally suppressed the expression of pro-inflammatory cytokines in dendritic cells including IL–6, TNFα and IL–12. In addition, the differentiation of Th1 cells, but not Th2, Th17 and regulatory T cells, was significantly hampered by lidocaine.

## Materials and Methods

### Ethics statement

All mouse experiments were performed as approved by Seoul National University Institutional Animal Care and Use Committee (Seoul National University approved protocol #SNU-140602-2-2).

### Mice

Female C57BL/6 mice at the age of six to ten weeks were purchased from Orient Bio (Gyeonggi, South Korea). OT-II TcR transgenic mice were bred in house with breeders derived from Jackson Laboratories (Maine, USA) and only six to ten weeks of age female mice were used. Mice were maintained under specific pathogen-free animal facility in sterile individual ventilation cages at the Seoul National University with free access to gamma-irradiated standard cereal-based diets (Zeigler) and sterile water. CO_2_ inhalation using gradual fill method was used as a method of euthanasia to minimize potential pain. For *in vivo* experiments, 3–4 mice per group were used. All *in vivo* experiments were repeated at least twice using protocols approved by Seoul National University Institutional Animal Care and Use Committee. Total 52 mice used were in the present study.

### Generation of bone marrow-derived dendritic cells

Bone marrow cells were obtained from femurs and tibia of wild type mice by flushing with PBS containing 1.5% fetal bovine serum (GenDepot). Red blood cells were lysed using ACK buffer (150 mM NH_4_Cl, 10 mM KHCO_3_, 0.1 mM EDTA). RBC-lysed cells were seeded on T75 flask in 20 ml of PRMI–1640 (Gibco) supplemented with 10% FBS, 2mM L-glutamine (Gibco), 100 U/ml penicillin (Gibco), 100 μg/ml streptomycin (Gibco), 55μM 2-mercaptoethanol (Gibco) and 10 μg/ml gentamycin (Gibco) with 10 ng/ml recombinant mouse GM-CSF (Peprotech). On day 1, non-adherent cells were removed. Half of the medium was replaced with fresh medium containing 10 ng/ml GM-CSF every 2–3 days. On day 7, loosely attached and non-adherent cells were collected and used as bone marrow-derived dendritic cells. In some experiments, dendritic cells were further enriched using CD11c microbeads (Miltenyi Biotec).

### Dendritic cell stimulation with TLR ligands

Bone marrow-derived dendritic cells were stimulated with either 100 ng/ml of LPS, 1 μg/ml poly(I:C) or R837 in the presence of various lidocaine concentrations indicated in the figure legend. Lidocaine stock solutions were prepared by dissolving the chemical in the EtOH. In all cases, the same amount of EtOH was used as a control. Bone marrow-derived dendritic cells were stimulated with TLR ligands in the presence or absence of lidocaine for 4 h and 24 h for quantitative real-time PCR analysis and cytokine ELISA, respectively.

### Cytokine ELISA

The amount of IL-12p70, TNF-α, IL–6, IL–10, IFNγ, IL–4, IL–5, IL–13 and IL–17 in the cultured supernatant were determined with an ELISA kit (all from Biolegend except IL–13 from eBioscience). All assays were performed according to the manufacturer’s protocol.

### Quantitative real-time PCR

Total RNA was obtained using TRIzol reagent (Invitrogen) and cDNA was synthesized with RevertAid reverse transcriptase and oligo(dT) primers (Thermo Scientific) [30]. Levels of mRNA expression of each gene were measured with 7500 Fast Real-Time PCR system (Applied Biosystems) using iTaq SYBR Green Supermix (Bio-Rad Laboratories). Data were normalized to the expression of *Gapdh*. The following primer pairs were used: *Il12a*, 5’-CCACCCTTGCCCTCCTAAAC–3’ and 5’-GGCAGCTCCCTCTTGTTGTG–3’; *Il12b*, 5’-CTTGCAGATGAAGCCTTTGAAGA–3’ and 5’-GGAACGCACCTTTCTGGTTACA–3’; *Il27*, 5’-CTCTGCTTCCTCGCTACCAC–3’ and 5’-GGGGCAGCTTCTTTTCTTCT–3’; *Il23*, 5’-AAGTTCTCTCCTCTTCCCTGTCGC–3’ and 5’-TCTTGTGGAGCAGCAGATGTGAG–3’; *Ebi3*, 5’-TCCCCGAGGTGCACCTGTTCTCC–3’ and 5’-GGTCCTGAGCTGACACCTGG–3’; *Il6*, 5’-TATGAAGTTCCTCTCTGCAAGAGA–3’ and 5’- TAGGGAAGGCCGTGGTT–3’; *Il1b*, 5’-AAGGAGAACCAAGCAACGACAAAA–3’ and 5’-TGGGGAACTCTGCAGACTCAAACT–3’; *Il10*, 5’-ATAACTGCACCCACTTCCCAGTC–3’ and 5’-CCCAAGTAACCCTTAAAGTCCTGC–3’; *Tnf*, 5’-GACGTGGAAGTGGCAGAAGAG–3’ and 5’-TGCCACAAGCAGGAATGAGA–3’; *Tbx21*, 5’-CAACAACCCCTTTGCCAAAG–3’ and 5’-TCCCCCAAGCAGTTGACAGT–3’; *Eomes*, 5’-TGAATGAACCTTCCAAGACTCAGA–3’ and 5’-GGCTTGAGGCAAAGTGTTGACA–3’; *Ifng*, 5’-GATGCATTCATGAGTATTGCCAAGT–3’ and 5’-GTGGACCACTCGGATGAGCTC–3’; *Gapdh*, 5’-GAGAACTTTGGCATTGTGG–3’ and 5’-ATGCAGGGATGATGTTCTG–3’.

### Western blot analysis

Raw264.7 cell lines were cultured in DMEM (Gibco) supplemented with 10% FBS, 100 U/ml penicillin, 100 μg/ml streptomycin and 10 μg/ml gentamycin. One day prior to treatment, 1 x 10^6^ cells/well were seeded on a 6-well plate. Cells were treated with 0, 0.2, 0.4, 0.8 mg/ml of lidocaine for 2 h and subsequently stimulated with LPS for 10 min. Cells were washed with cold PBS, lysed with NP–40 lysis buffer containing protease inhibitor cocktail (GenDepot) and incubated with continuous agitation at 4°C for 30 min. After centrifugation at 13,000 g for 15 min, supernatants were taken and 30 μg of protein were used for SDS-PAGE. Protein samples were transferred to Immobilon-P PVDF membrane (Millipore). The following antibodies were used for western blot analysis: anti-IκB-α (L35A5, 1:1000 dilution, Cell Signaling Technology), anti-β-actin (AC–15, 1:5000 dilution, Abcam), anti-mouse IgG-HRP (sc–2005, 1:5000 dilution, Santa Cruz Biotechnology).

### Helper T cell differentiation *in vitro*

Naïve CD4^+^ T cells were sorted from spleen and lymph nodes of wild type mice as CD4^+^CD62L^+^CD25^-^CD44^-^ population with FACSAria III cell sorter (BD BioScience) as previously described [30, 31]. Bone marrow-derived dendritic cells were purified as CD11c^+^ population with CD11c microbead. Naïve CD4^+^ T cells (1 x 10^5^/well) and CD11c^+^ dendritic cells (1 x 10^4^/well) were co-cultured in the presence of soluble anti-CD3 (0.3 μg/ml) (145-2C11, BioXcell) for 4 days. For Th1 differentiation, cells were treated with LPS (100 ng/ml) (Sigma). For Th2 differentiation, IL–2 (10 ng/ml) (eBioscience), IL–4 (10 ng/ml) (Peprotech), anti-IFNγ (XMG1.2, 5 μg/ml) were added [31]. For Th17 differentiation, cells were stimulated with LPS (100 ng/ml) and TGF-β (3 ng/ml) (Peprotech) [30]. For regulatory T differentiation, cells were cultured with TGF-β (5 ng/ml). For APC-free Th1 cell differentiation, plates were coated with anti-CD3 (1 μg/ml) and anti-CD28 (2 μg/ml) (37.51, BioXcell) overnight at 4°C. 1 x 10^5^ naïve CD4^+^ T cells were stimulated with IL–2 (2 ng/ml) and IL–12 (10 ng/ml) (Peprotech) or supernatant of dendritic cells stimulated with LPS in the presence or absence of lidocaine for 4 days. *In vitro* differentiated CD4^+^ T cells were incubated with 100 ng/ml PMA (Sigma) and 1 μM ionomycin (Sigma) in the presence of Brefeldin A and Monesin (Both from eBioscience) for 4 h.

### Flow cytometry

Cell surface molecules were stained in PBS containing 1.5% FBS for 30 min at 4°C. Subsequently, cells were fixed with fixation buffer (eBioscience) for 30 min at 4°C and washed with permeabilization buffer (eBioscience). For Foxp3 staining, a Foxp3 staining kit (eBioscience) was used according to the manufacturer’s protocol. Intracellular cytokines and transcription factor were stained in the permeabilization buffer. The following antibodies were used: Alexa488 or PerCP-Cyanine5.5-conjugated anti-IFNγ (XMG1.2, Biolegend), Pacific Blue-conjuated anti-Foxp3 (MF–14, Biolegend), FITC-conjugated anti-TCR Vα2 (B20.1, Biolegend), PE-conjugated anti-IL–17 (TC11-18H10.1, Biolegend), PE-Cyanine7-conjugated anti-CD44 (IM7, Biolegend), PerCP-Cyanine5.5 or APC-Cyanine7-conjugated anti-CD4 (GK1.5, Biolegend), Alexa647-conjugated anti-IL–4 (11B11, Biolegend) and APC-conjugated anti-IL–5 (TRFIC5, Biolegend). Cells were analyzed with FACSCalibur, FACSVerse or FACSAria III flow cytometer (BD BioScience). Obtained data were analyzed with FlowJo software.

### Dendritic cell transfer study

Bone marrow-derived dendritic cells were resuspended at 1.5 x 10^6^ cells/ml in RPMI–1640 medium with 2% FBS and pulsed with 1 μg/ml of ovalbumin peptide (OVA_323-339_) in the presence of 0.4 mg/ml of lidocaine or vehicle for 2 h followed by being stimulated with 100 ng/ml LPS for 1h. Cells were washed with PBS for three times and re-suspended in PBS before being intravenously injected into OT-II mice (5 x 10^4^ cells/injection). Four days after injection, splenic lymphoid cells from the recipient mice were obtained and restimulated with 2 μg/ml of OVA_323-339_ for 48 h. The amounts of IFNγ, IL–17 and IL–4 in the supernatant were measured by ELISA. Lymphoid cells from peripheral lymph node (inguinal, brachial, axillary and cervical nodes) were stimulated with PMA and ionomycin in the presence of monesin and brefeldin A for 4 h and analyzed for intracellular cytokine staining by flow cytometer.

### Ovalbumin-alum immunization

Immunization was performed according to the manufacturer’s protocol. In brief, 1:1 mixture of Imject alum (ThermoFisher Scientific) and 50 μg of ovalbumin (Sigma-Aldrich) were injected intraperitoneally on day 0. One mg of lidocaine or ethanol as a vehicle was injected intraperitoneally every other day for total three times. Seven days after Ovalbumin immunization, splenic lymphoid cells were obtained and restimulated with indicated dose of ovalbumin for 72 h. The levels of IL–5 and IL–17 in the supernatant were analyzed by ELISA.

### Statistics

Data were analyzed with GraphPad Prism 6 software (GraphPad Software). Two-tailed student’s t test was used to determine statistical significance. P values less than 0.05 were considered statistically significant.

## Results and Discussion

### Differential regulation of LPS-induced expression of cytokines in dendritic cells by lidocaine

Dendritic cells play an essential role in bridging innate and adaptive immunity. To determine if the function of dendritic cells is affected by lidocaine, we analyzed the expression of cytokines in dendritic cells upon stimulation with LPS in the presence or absence of lidocaine. Cytokines in IL–12 family, IL–6 and IL–1β are produced by dendritic cells upon PAMPs and DAMPs stimuli and they play crucial roles in shaping the effector functions of T cells that recognize cognate antigens presented on dendritic cells [32–35]. LPS is known to induce the expression of all IL–12 family cytokines in dendritic cells and macrophages [36].

**Fig 1:**
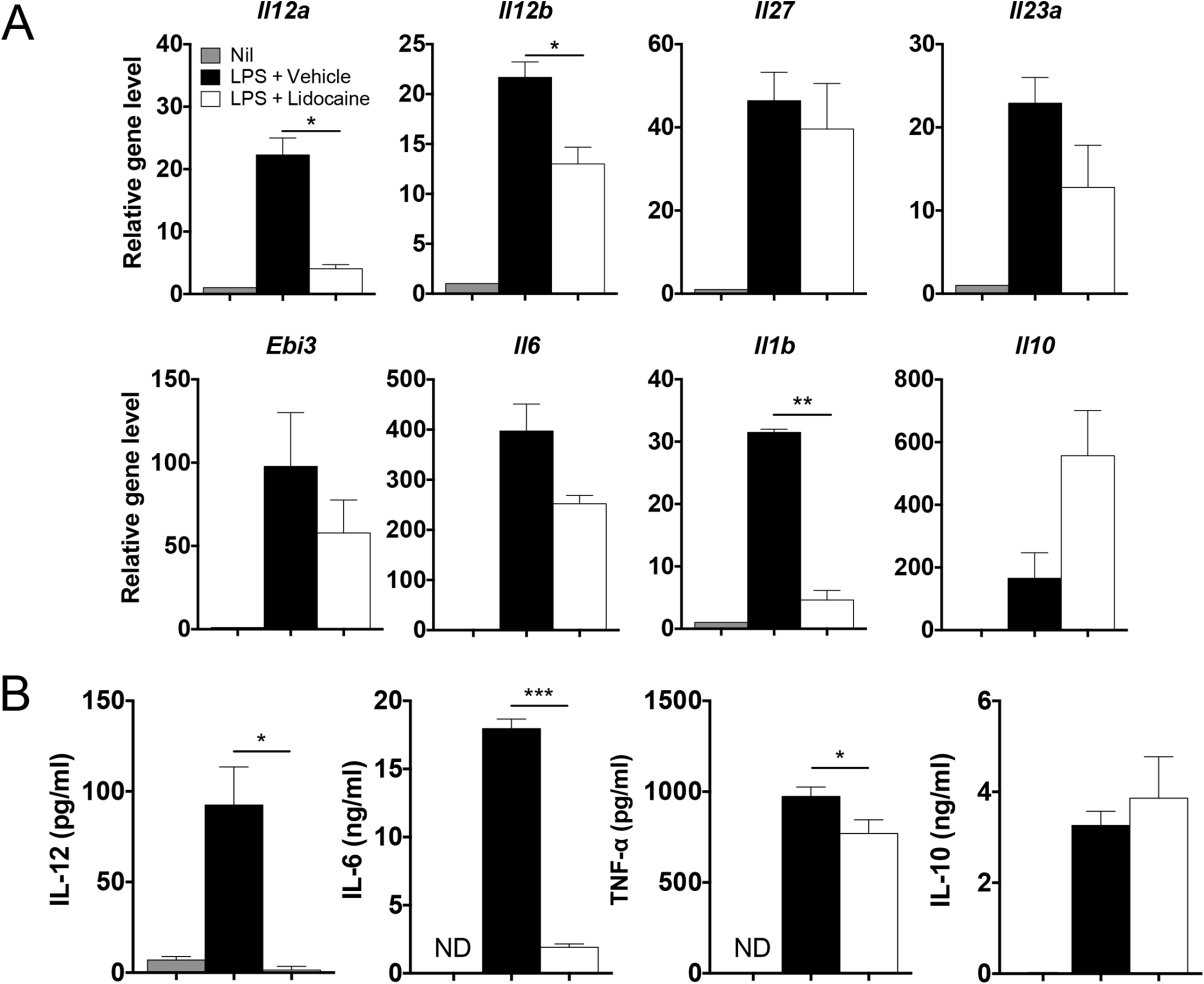
Effects of lidocaine on the expression of various cytokines upon LPS stimulation. Bone marrow-derived dendritic cells were stimulated with 100 ng/ml of LPS in the presence of vehicle (EtOH) or 0.2 mg/ml lidocaine for 4 h and 24 h to examine mRNA expression and cytokine production, respectively. (A) The mRNA levels of the indicated genes were analyzed by quantitative RT-PCR. (B) The amounts of each cytokine produced were measured by ELISA. All experiments were performed at least three times. Data shown are mean ± SEM. *p<0.05; ***p<0.001; ND, not detected.

When bone marrow-derived dendritic cells were stimulated with LPS, the expression of *Il12a*, *Il12b*, *Il27*, *Il23a*, *Ebi3*, *Il6*, *Il1b*, *and Il10* transcripts were all remarkably induced within 4 hours. As depicted in Fig 1A, the expression levels of *Il12a*, *Il12b* and *Il1b* were significantly decreased by the addition of lidocaine, whereas those of *Il23a* and *Il6* were only slightly decreased by the same treatment. Although it did not reach statistical significance, the expression level of *Il10* was further increased by lidocaine in LPS-stimulated dendritic cells. (Fig 1A). Consistently, the amounts of IL–12, IL–6 and TNFα produced by dendritic cells were decreased while IL–10 production was slightly increased by lidocaine (Fig 1B).

**Fig 2:**
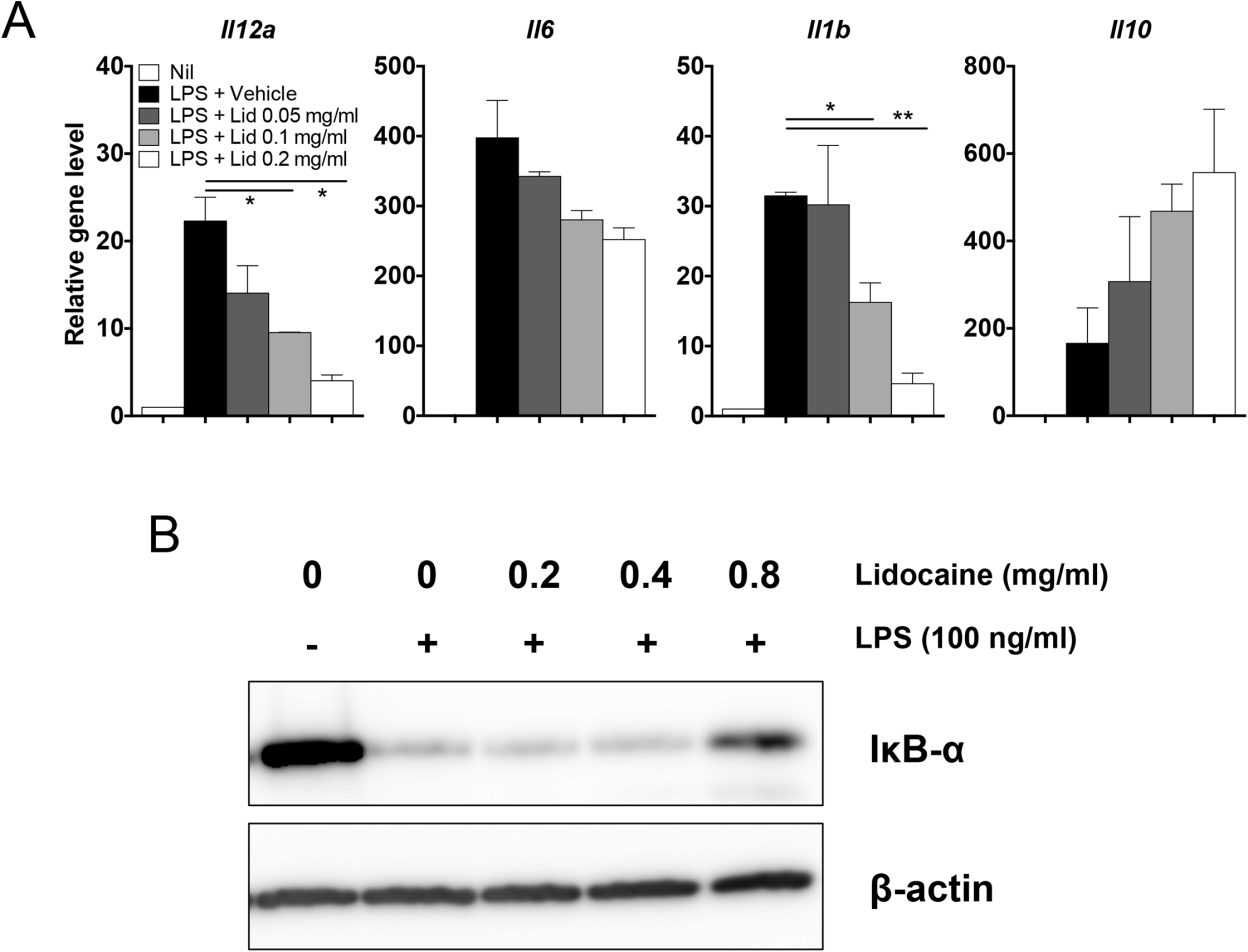
Lidocaine regulates the expression of cytokines and NF-κB signaling pathway in a dose-dependent manner. (A) Bone marrow-derived dendritic cells were stimulated with 100 ng/ml of LPS together with increasing concentrations of lidocaine for 4 h. The mRNA levels of the indicated genes were analyzed by quantitative RT-PCR. (B) Raw 264.7 cells were treated with increasing doses of lidocaine for 2 h and stimulated with LPS for 20 min. The expression of IκB-α was examined by western blot. All experiments were performed at least three times. Data shown are mean ± SEM. *p<0.05; **p<0.01.

To further characterize the immune modulatory effects of lidocaine on the activation of dendritic cells, increasing doses of lidocaine were added into LPS-stimulated dendritic cells. The levels of LPS-induced *Il12a*, *Il6* and *Il1b* transcripts were all decreased by lidocaine in a dose-dependent manner, while the reverse was true for *Il10* (Fig 2A). It is notable that the lidocaine-mediated suppression of *Il12a* and *Il1b* seemed more sensitive than that of *Il6*. Since the expression of these genes in antigen-presenting cells after LPS stimulation depends on the activation of NF-κB pathway [37], we next determined if lidocaine inhibits the degradation of IκB and found that lidocaine inhibited the degradation of IκB in a dose-dependent manner (Fig 2B). Together, these data demonstrate that lidocaine differentially regulates the expression of pro- and anti-inflammatory cytokines in dendritic cells stimulated with LPS *in vitro*.

### Effects of lidocaine on cytokine expression in response to diverse toll-like receptor ligands

Diverse toll-like receptor signals not only promote immune defense against infectious agents, but also mediate the development of autoimmunity [38, 39]. Since lidocaine modulated LPS-stimulated production of cytokines in dendritic cells, we next sought to determine if lidocaine could also regulate the cytokine expression induced by other types of toll-like receptor ligands. To this end, we stimulated bone marrow-derived dendritic cells with LPS, poly(I:C) and R837 as ligands for TLR4, TLR3 and TLR7, respectively [40].

**Fig 3:**
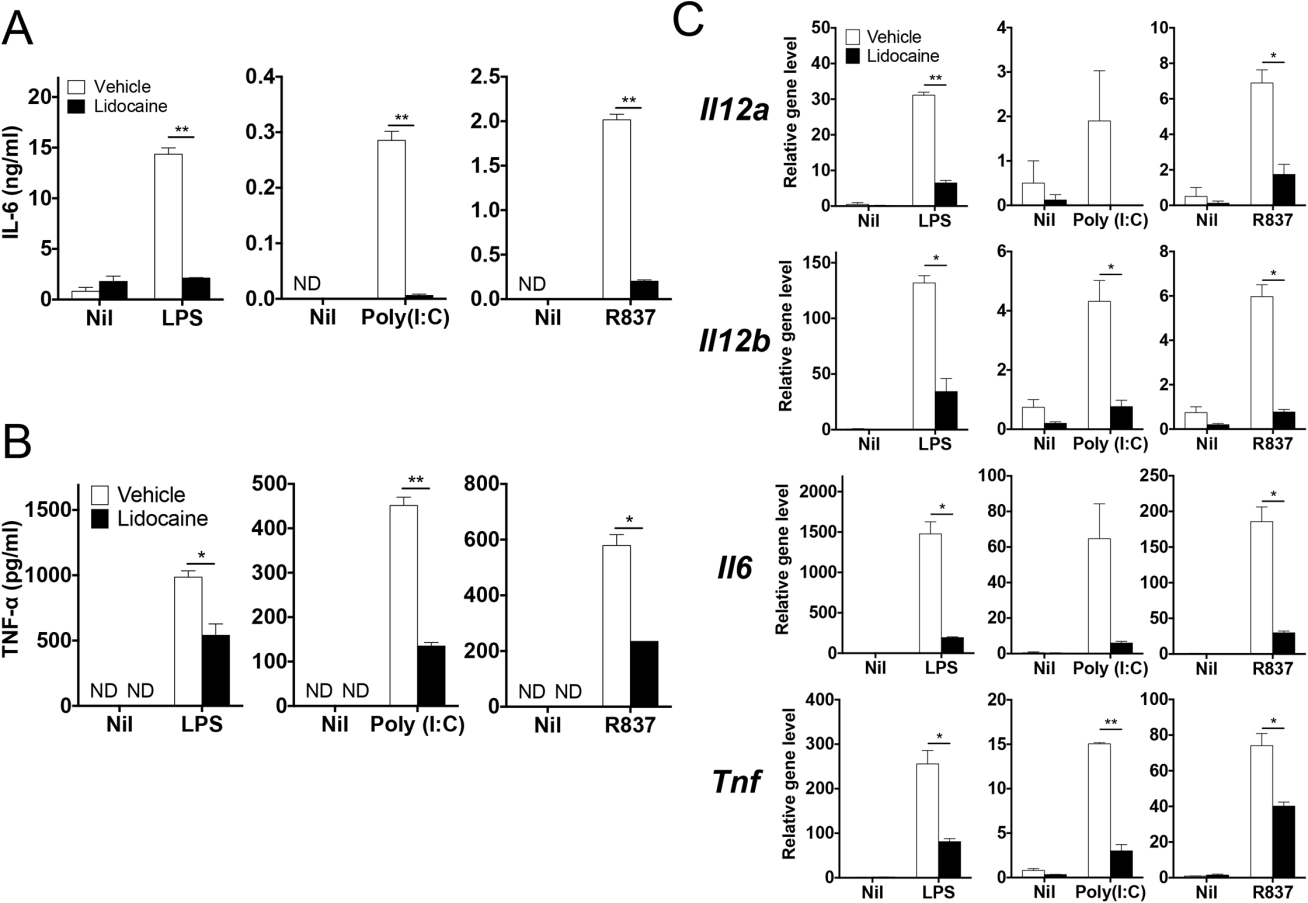
Regulation of cytokines expression in dendritic cells in response to various TLR ligands by lidocaine. (A & B) Bone marrow-derived dendritic cells were stimulated with LPS (100 ng/ml), poly(I:C) (1 μg/ml) or R837 (1 μg/ml) in the presence of lidocaine (0.4 mg/ml) or vehicle. The amounts of IL–6 and TNF-α in the supernatant were measured by ELISA. (C) The mRNA levels of the indicated genes were analyzed by quantitative RT-PCR. Data represent at least two independent experiments. Data shown are mean ± SEM. *p<0.05; **p<0.01; ND, not detected.

Again, lidocaine significantly inhibited the levels of *Il6*, *Il12a* and *Tnf* induced by LPS, which was consistently supported by the reduction in the amounts of IL–6 and TNFα by the same treatment (Fig 3A–3C). Stimulation of dendritic cells with poly(I:C) induced the expression of *Il12a*, *Il12b*, *IL6* and *Tnf*; however, addition of lidocaine remarkably decreased the levels of these transcripts as well as the amounts of IL–6 and TNFα (Fig 3A–3C). Similarly, the upregulated expression of *Il6* and *Il12a* as well as the production of IL–6 and TNFα induced by R837 were significantly attenuated by the addition of lidocaine (Fig 3A–3C). Thus, lidocaine appeared to suppress the TLR3-, 4- and 7-stimulated expression of cytokines in dendritic cells including IL–6, IL–12 and TNFα.

### Effects of lidocaine on helper T cell differentiation *in vitro*

The decreased production of IL–6 and IL–12 family cytokines in dendritic cells by lidocaine prompted us to determine whether lidocaine impacts helper T cell differentiation from naïve T cells. Cytokines such as IL–12, IL–6, IL–4 and TGF-β produced by innate immune cells drive the differentiation of Th1, Th17 Th2 and regulatory T cell lineages by stimulating STAT4, STAT3, STAT6, STAT5 respectively [41].

**Fig 4:**
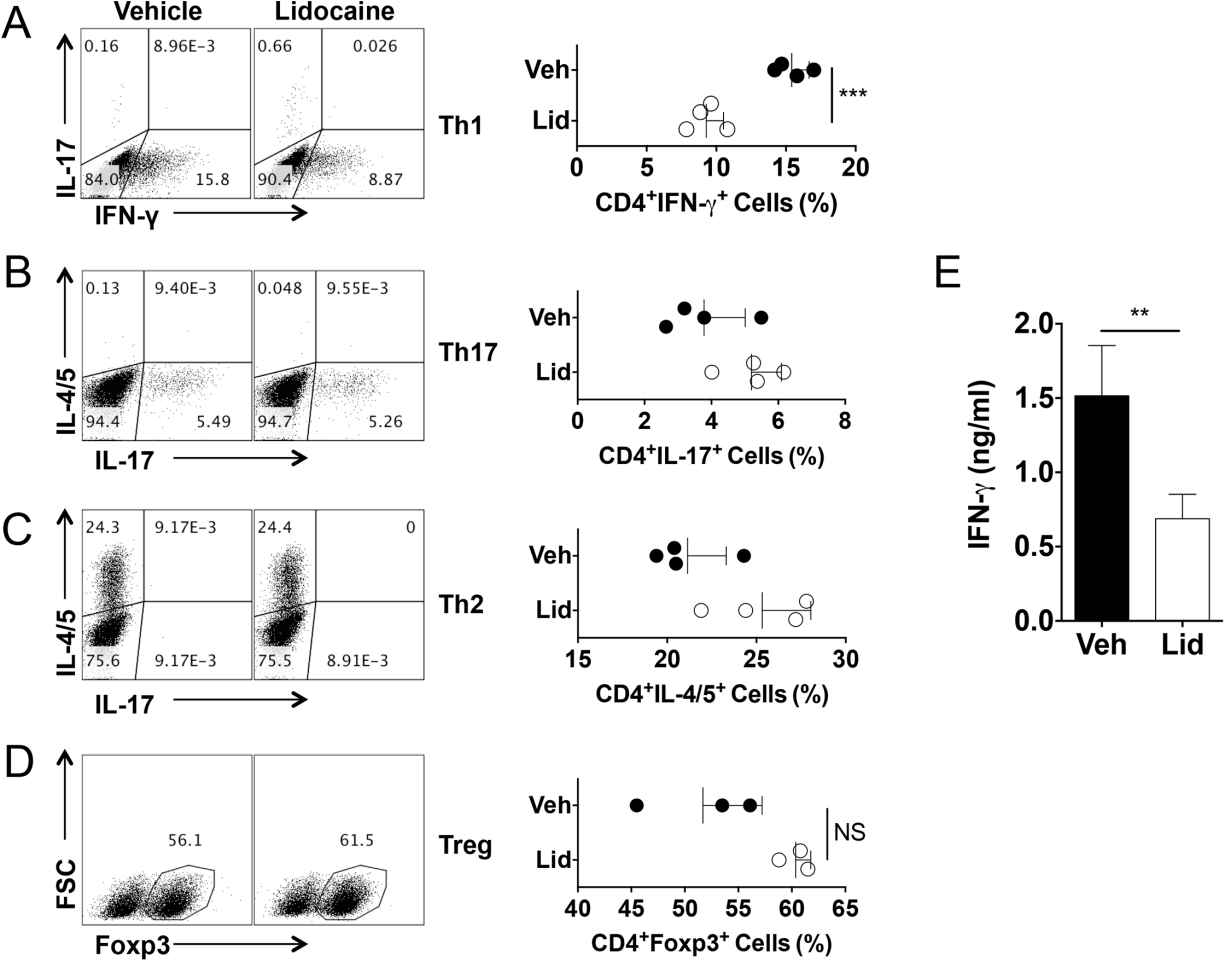
Lidocaine inhibits dendritic cell-mediated Th1 cell differentiation while having little effects on dendritic cell-mediated Th2, Th17, regulatory T cell differentiation *in vitro*. Naïve CD4^+^ T cells were co-cultured with bone marrow-derived dendritic cells with Th1, Th17, Th2 or regulatory T cell differentiation conditioned-media or cultured with plate-coated anti-CD3 and anti-CD28 with supernatant of dendritic cells stimulated with LPS in the presence of lidocaine (0.2 mg/ml or indicated dose) or vehicle. (A-D) The frequencies of IFN-γ, IL–17, IL–4/5 or Foxp3 positive cells among CD4^+^ population were measured by flow cytometer. (E) The level of IFN-γ was measured using co-cultured supernatants from Th1 differentiation condition. Data represent at least two independent experiments. Data shown are mean ± SEM. *p<0.05; **p<0.01; ***p<0.001; NS, not significant.

To determine if the differentiation of each helper T cell subset is regulated by lidocaine, we employed a well-established *in vitro* T cell differentiation system. For Th1 cell differentiation, naïve CD4^+^ cells were co-cultured with bone marrow-derived dendritic cells together with anti-CD3 and LPS in the presence or absence of lidocaine. As shown in Fig 4A, lidocaine treatment significantly decreased the frequencies of IFNγ-producers. By contrast, the same treatment resulted in a slight increase in the frequency of IL-17-producers among CD4^+^ cells compared to vehicle treatment in this Th1 cell-skewing condition (vehicle vs. lidocaine; 0.148 ± 0.0075 vs. 0.7175 ± 0.0962; *p*<0.05). As a result, the production of IFNγ was also significantly lowered by lidocaine (Fig 4E).

For Th17 cell differentiation, naïve CD4^+^ T cells were stimulated with bone marrow-derived dendritic cells in the presence of anti-CD3 together with LPS and TGF-β [35, 42]. The frequencies of IL-17-producers among CD4^+^ T cells were comparable between vehicle- and lidocaine-treated groups (Fig 4B). Hence, the increased frequency of Th17 cells in the Th1-polarizing condition (Fig 4A) was probably due to the reduced Th1 cell differentiation rather than the direct effect of lidocaine on Th17 cell differentiation.

For Th2 cell differentiation, naïve CD4^+^ T cells were stimulated by using a similar culture condition except the addition of IL–4 instead of LPS and TGF-β [31]. The frequencies of IL–4 or IL–5 producers among CD4^+^ T cells were also comparable between the two groups (Fig 4C).

For regulatory T cell differentiation, naïve CD4^+^ T cells were cultured with bone marrow-derived dendritic cells in the presence of TGF-β. Lidocaine had little effect on the frequency of Foxp3^+^ cells among CD4^+^ T cells (Fig 4D).

Overall, the addition of lidocaine had little effects on the differentiation of Th17 cell, Th2 cell and regulatory T cell while it inhibited the dendritic cell-mediated differentiation of Th1 cell differentiation *in vitro* in this experimental setting.

### Effects of lidocaine on dendritic cell-mediated Th1 cell differentiation *in vitro*

**Fig 5:**
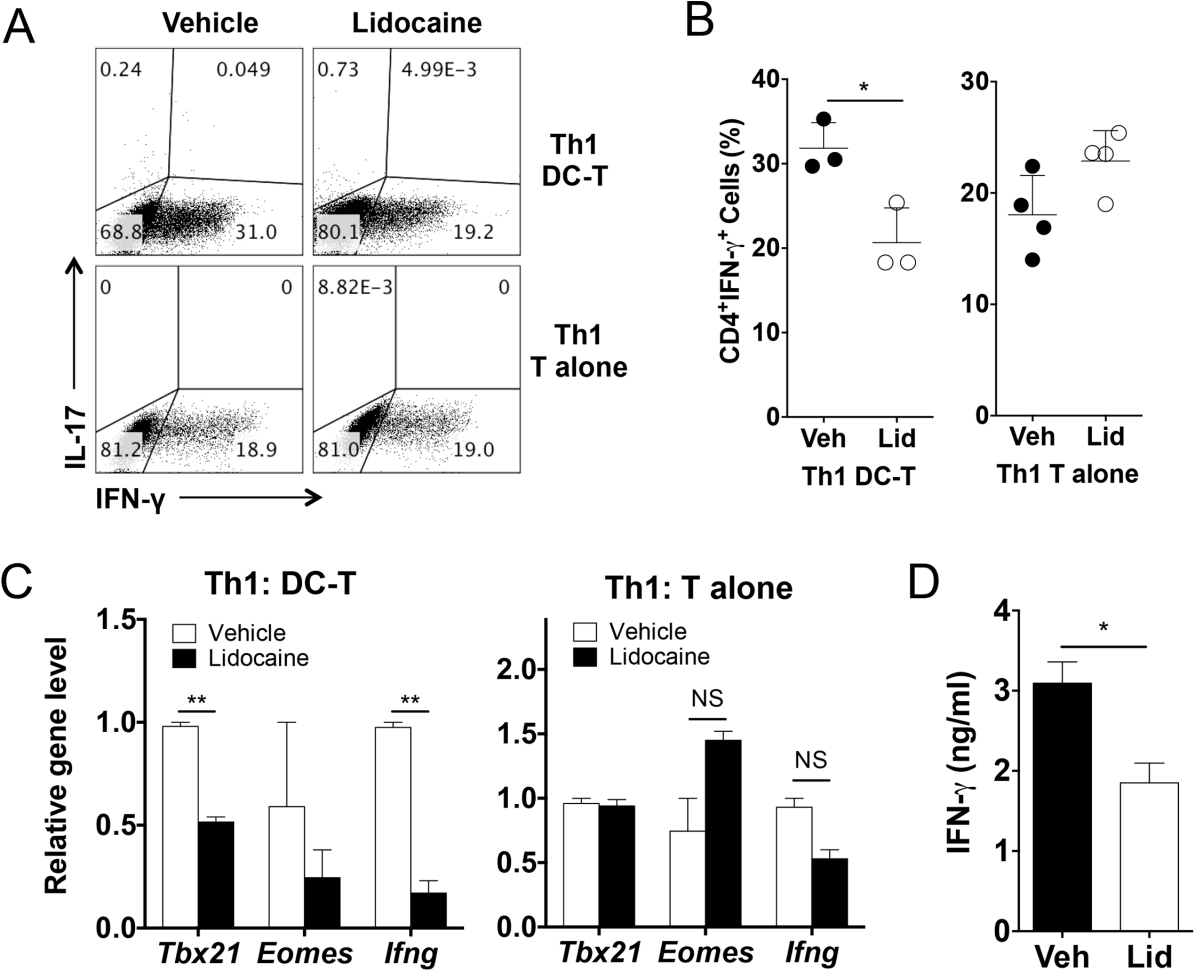
Lidocaine inhibits dendritic cell-mediated Th1 cell differentiation *in vitro*. Naïve CD4^+^ T cells were either co-cultured with bone marrow-derived dendritic cells in the presence of soluble anti-CD3 and LPS, or in anti-CD3, CD28 pre-coated plates in the presence of IL–2 and IL–12 for Th1 cell differentiation. Lidocaine was added at a concentration of 0.2 mg/ml. (A & B) The frequencies of IFNγ or IL–17 producing cells among CD4^+^ T cells. (C) The mRNA levels of the indicated genes. (D) The levels of IFN-γ in the cultured supernatants of naïve CD4^+^ T cells cultured with vehicle- or lidocaine-conditioned media. Data represent at least three independent experiments. Data shown are mean ± SEM. **p<0.01; ***p<0.001; NS, not significant.

To further dissect if the negative effect of lidocaine on Th1 cell differentiation is due to its effect on dendritic cell or on T cells, we utilized a dendritic cell-free Th1 cell differentiation system in which naïve CD4^+^ T cells were stimulated with plate bound anti-CD3 and anti-CD28 in the presence of recombinant IL–12. Of note, we observed that the addition of lidocaine did not change the frequency of IFNγ-producers in dendritic cell-free condition while reducing the frequency of IFNγ-producers in DC/T cell-coculture condition (Fig 5A and 5B). In addition the levels of *Tbx2*1 and *Ifng* transcripts in the stimulated T cells under Th1-skewing condition did not change in dendritic cell-free condition, however, the levels of these transcripts were significantly decreased by lidocaine in DC/T cell-coculture condition (Fig 5C).

We next assessed whether the suppression of Th1 cell differentiation is due to soluble proteins from dendritic cells or direct contact between dendritic cells and T cells. Cultured supernatant of LPS stimulated dendritic cells in the presence of lidocaine or vehicle was harvested and added into the naïve T cell culture stimulated with plate-coated anti-CD3 and anti-CD28. As shown in Fig 5D, the levels of IFNγ produced by the stimulated T cells were decreased when cultured in the lidocaine-conditioned media compared with vehicle-conditioned media. Given that lidocaine suppressed the production of IL–12, these results together strongly suggest that lidocaine inhibits the differentiation of Th1 cells *in vitro*, presumably by modulating the production of Th1-promoting cytokines from dendritic cells rather than directly affecting T cells.

### Regulation of antigen-specific Th1 cell responses by lidocaine *in vivo*

The modulation of Th1 cell differentiation *in vitro* by lidocaine prompted us to examine if lidocaine also impacts antigen-specific Th1 cell responses *in vivo*. To this end, bone marrow-derived dendritic cells were pulsed with OVA_323-339_ and were additionally stimulated with LPS in the presence or absence of lidocaine before they were adoptively transferred into OT-II mice whose transgenic T cell receptor recognizes OVA_323-339_ in the context of MHC class II. Lymphoid cells from the recipient mice were analyzed for the production of effector cytokines.

**Fig 6:**
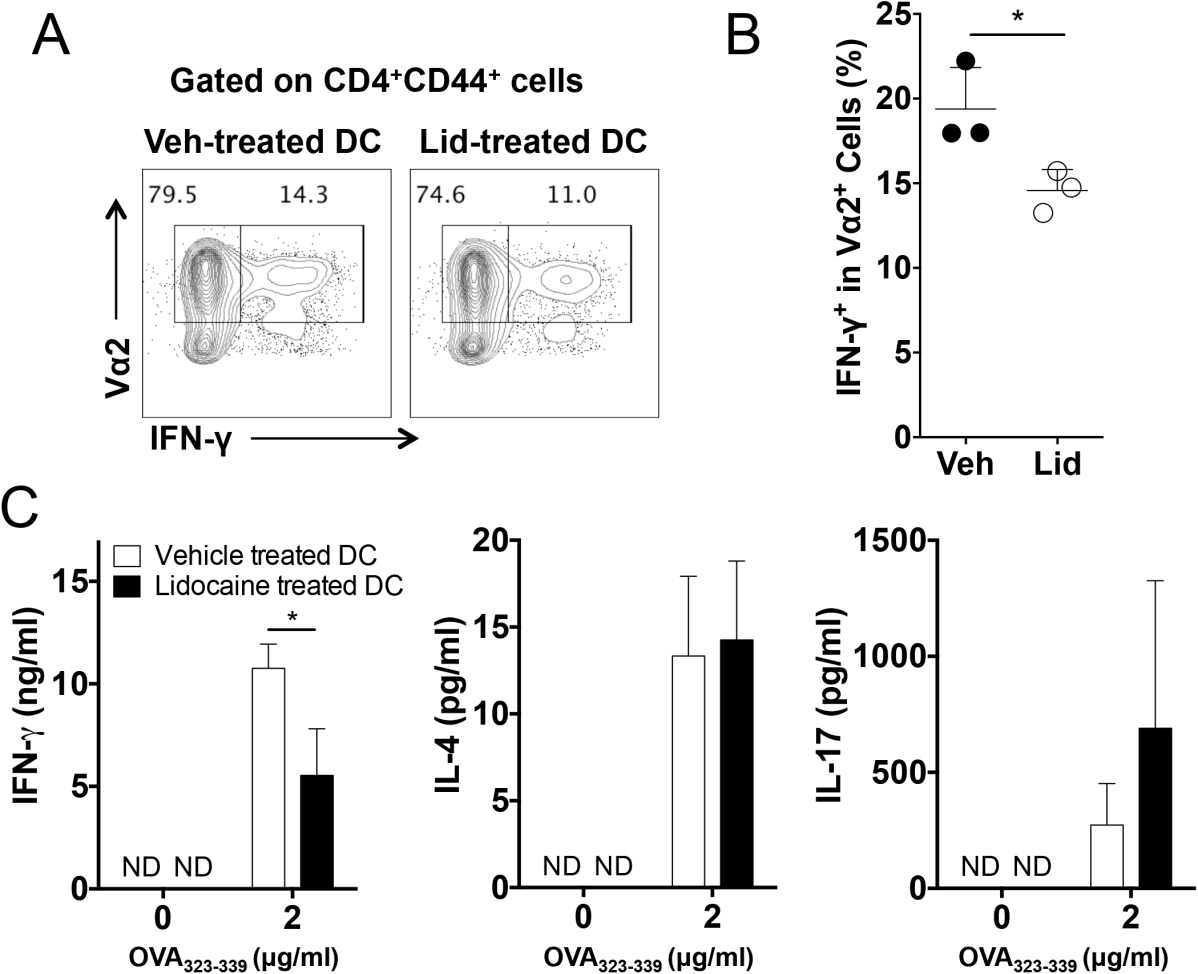
Inhibition of dendritic cell-mediated antigen-specific Th1 cell responses by lidocaine *in vivo*. Bone marrow-derived dendritic cells were pulsed with OVA_323-339_ in the presence of lidocaine or vehicle before being transferred into OT-II TcR transgenic mice (n = 3~4). (A and B) The frequencies of IFN- γ producers among Vα2^+^ cells. (C) The amounts of the indicated cytokines in the supernatant of splenocyte stimulated with OVA_323-339_ were measured by ELISA. Data represent two independent experiments. Data shown are mean ± SEM. *p<0.05.

Compared with the recipients of vehicle-treated dendritic cells, the frequency of IFNγ-producers among CD44^hi^Vα2^+^ cells was moderately but significantly decreased in the recipient of lidocaine-treated dendritic cells (Fig 6A and 6B). In conjunction with the reduced frequency of IFN-γ producers, the levels of antigen-induced IFNγ were significantly diminished in the latter group (Fig 6C). By contrast, the levels of IL–4 and IL–17 showed no significant difference between the two groups, suggesting that the antigen-specific Th2 and Th17 cell responses remained comparable (Fig 6C).

To further address the role of lidocaine in antigen-specific Th2 responses *in vivo*, groups of mice were immunized with OVA in alum suspension and were additionally administered with vehicle or lidocaine. The amounts of IL–5 as well as IL–17 were comparable in the supernatant of OVA-restimulated splenocytes between vehicle- and lidocaine-treated groups (S1 Fig). These results indicate that lidocaine specifically inhibits the development of antigen-specific Th1 cell responses induced by dendritic cells *in vivo*, while it has little effects on Th2 cell and Th17 cell responses.

Understanding the immune modulatory functions of anesthetics is important because it may affect the host defense immunity in patients undergoing surgery. Intravenous lidocaine is widely used for postoperative analgesia. In the present study, we aimed to determine the potential immune regulatory function of lidocaine on the differentiation of diverse helper T cell lineages as well as in the activation of dendritic cells. Our results demonstrate that lidocaine dampens the production of pro-inflammatory cytokines from dendritic cells upon TLR stimulation and suppresses the differentiation of Th1 cells *in vitro* as well as *in vivo*. Since DAMP ligands such as HMGB1 also signal through NFκB to induce relevant cytokines [43], it seems that lidocaine also negatively regulates the inflammatory functions of DAMP signals, possibly through the negative regulation of NF-kB signaling pathway. Further studies are needed to clearly define the detailed molecular mechanisms of how lidocaine impacts cytokine production in dendritic cells. Considering that IL–12 and Th1 cell responses play an essential role in the clearance of intracellular pathogens and in anti-tumor immunity [44], our findings propose that there should be a serious consideration when we choose the type of anesthetics in patients with cancer and infectious diseases, since certain anesthetic agents such as lidocaine potentially incapacitate host defense immunity against such diseases.

Administration of lidocaine is known to induce allergic reactions in some patients [28, 29], suggesting that it may trigger Th2 cell responses. In our experimental system, however, we observed that lidocaine had little role in Th2 cell differentiation *in vitro* as well as *in vivo*. The Th2 cell differentiation system used in this study required exogenous IL–4 since this cytokine is generally not produced by dendritic cells. Hence, it is still possible that lidocaine can influence Th2 cell responses in patients by regulating the production of IL–4 or other Th2 cell-promoting cytokines such as IL–25, IL–33 and TSLP. In addition, IL–12 and Th1 cell cytokine IFNγ are well known to strongly inhibit Th2 cell responses [45]. Therefore, lidocaine may provide a Th2 cell favorable environment by inhibiting the production of IL–12 and IFNγ from dendritic cells and T cells. Further studies will be needed to dissect the *in vivo* function of lidocaine in Th2 cell immunity.

IL–6 provides crucial signal to T cells during Th17 cell differentiation. Even though lidocaine strongly inhibited the production of IL–6 from dendritic cells, it did not affect the differentiation of Th17 cell *in vitro* and *in vivo*. In addition, our quantitative RT-PCR analysis showed that the production of IL–23 and IL–1β, which promote Th17 maturation [35, 46], was likely suppressed by lidocaine. Therefore it is not clear why the differentiation of Th17 cells was intact in the presence of lidocaine. One possible explanation is that lidocaine may also inhibit cytokines that negatively regulate Th17 cell responses. For instance, IL–27 inhibits Th17 cell responses [47], and the expression of *Ebi3* transcript was significantly decreased by lidocaine. IL–2 is another cytokine that can suppress Th17 cell differentiation via the activation of STAT5 [48]. In this regard, lidocaine has been recently shown to inhibit the production of IL–2 from Jurkat T cells [49]. Thus it is possible to surmise that lidocaine also down-regulates these negative regulators of Th17 cell differentiation, and thereby T cells might have enough signal for Th17 cell differentiation with reduced levels of IL–6, IL–23 and IL–1β.

In summary, our findings unveil that lidocaine can impact the differentiation of helper T cells into diverse lineage by modulating the production of cytokines from dendritic cells.

## Conclusion

The present study demonstrates that lidocaine inhibits the expression of IL–6, TNFα and IL–12 family cytokines while increasing that of IL–10 in dendritic cells upon stimulation with diverse TLR ligands. Addition of lidocaine appeared to significantly inhibit the dendritic cell-mediated differentiation of Th1 cells while it had little effects on the differentiation of Th17, Th2 and regulatory T cells. Collectively, our findings suggest that lidocaine can modulate the Th1 cell inducing capacity of dendritic cells.

## Supporting Information

S1 Fig Lidocaine has little effects on the production of Th2 or Th17 cell cytokines in mice immunized with ovalbumin in alum. Mice were immunized with Ovalbumin in alum on day 0 and were additionally given lidocaine or vehicle i.p. every other day for six days (n = 3~4). The levels of indicated cytokines in the supernatant of splenocytes restimulated with indicated concentration of ovalbumin were measured by ELISA. Data represent two independent experiments. Data shown are mean ± SEM.
(TIFF)
