# Supplementary figures and images for: Modulation of Dendritic Cell Activation and Subsequent Th1 Cell Polarization by Lidocaine
Source: PLoS One. 2015 Oct 7;10(10):e0139845. doi: 10.1371/journal.pone.0139845 (PMC4596553; doi:10.1371/journal.pone.0139845)

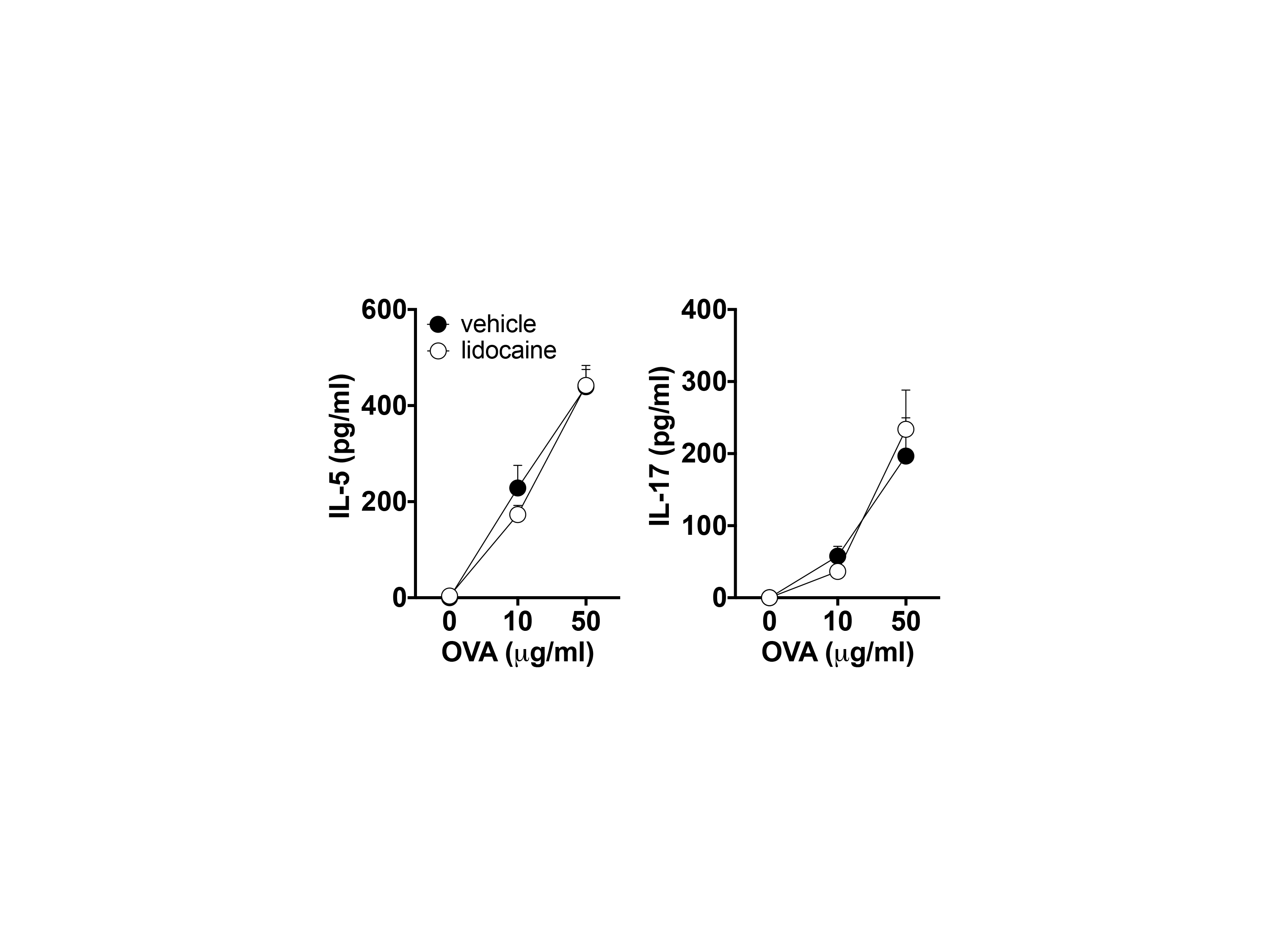

Supplement: S1 Fig — Mice were immunized with Ovalbumin in alum on day 0 and were additionally given lidocaine or vehicle i.p. every other day for six days (n = 3~4). The levels of indicated cytokines in the supernatant of splenocytes restimulated with indicated concentration of ovalbumin were measured by ELISA. Data represent two independent experiments. Data shown are mean ± SEM. (TIFF) [file pone.0139845.s001.tiff]
